# Surgery for massive splenomegaly

**DOI:** 10.1002/bjs5.1

**Published:** 2017-04-06

**Authors:** J. Lemaire, A. Rosière, C. Bertrand, B. Bihin, J. E. Donckier, L. A. Michel

**Affiliations:** ^1^ Surgical Services Université de Louvain – Medical School at Mont‐Godinne University Hospital Yvoir Belgium; ^2^ Internal Medicine Services Université de Louvain – Medical School at Mont‐Godinne University Hospital Yvoir Belgium; ^3^ Biostatistics Unit Université de Louvain – Medical School at Mont‐Godinne University Hospital Yvoir Belgium

## Abstract

**Background:**

Splenectomy for massive splenomegaly (spleen weight more than 1·5 kg) is commonly believed to be hazardous and to provide poor palliation. The aim of this cohort study was to investigate these issues and examine the many definitions of massive splenomegaly to see whether a better tool might be proposed for preoperative evaluation of these patients.

**Methods:**

Morbidity and long‐term outcomes were assessed in consecutive patients. Relief of pressure–volume‐related symptoms and sustainable independence from transfusion in patients were used to ascertain the impact of splenectomy.

**Results:**

Splenectomy was performed in 56 patients, mainly for non‐Hodgkin's lymphoma and myeloproliferative diseases. Median spleen weight was 2·3 (range 1·5–6·0) kg. Mortality at 180 days was zero, and the postoperative complication rate was 25 per cent (17 complications in 14 patients). At 2 years, relief of pain was maintained in 33 of 34 patients, with sustained independence from transfusion in 15 of 19 patients with anaemia and nine of 11 with thrombocytopenia. Spleen weight correlated negatively with BMI (P = 0·036).

**Conclusion:**

Splenectomy for massive splenomegaly is safe and provides effective palliation. Provisional cut‐off points relating to spleen size and BMI help to identify patients benefiting from a splenectomy, even those in a critical state.

## Introduction

The definition of massive splenomegaly, as proposed by Goldstone^1^ in 1978 and adopted by Bickerstaff and Morris^2^ in 1987, is a spleen weighing more than 1·5 kg, or about ten times the normal weight. Regardless of cause, splenomegaly of this magnitude may be symptomatic for physical reasons (pain caused by the weight and volume of the spleen), related to anaemia or thrombocytopenia, or follow splenic infarction. Splenectomy in these patients is widely believed to be hazardous and to provide only poor palliation. Observational studies^1^, ^2^, ^3^, ^4^, ^5^, ^6^, ^7^, ^8^ have reported mortality rates of 1·8–15 per cent and complication rates of 9–51 per cent. Numerous case reports and small series^9^, ^10^, ^11^, ^12^, ^13^, ^14^, ^15^, ^16^ have shown the feasibility of laparoscopic splenectomy for massive splenomegaly, but, as with open splenectomy, the criterion of a spleen weight greater than 1·5 kg has not been used consistently to refer to massive splenomegaly^3^, ^17^, ^18^. Some reports^9^ regarding the safety of laparoscopic approaches have included splenic specimens of only 500 g, whereas others^10^, ^11^, ^12^, ^13^ have included spleens of more than 600 or 1000 g. Other authors^14^, ^15^, ^16^ have categorized spleens weighing 600–1600 g as ‘massive’, and those weighing more than 1600 g as ‘supramassive’.

The aims of the present observational study were to examine the mortality and complications associated with splenectomy for massive splenomegaly (above 1·5 kg) and to see whether the procedure provided symptom palliation of worthwhile duration. It was hoped that these results might also identify splenic features and patient characteristics that could be used to simplify the terminology used for splenic surgery, particularly in the laparoscopic era.

## Methods

Consecutive patients with massive splenomegaly who underwent elective splenectomy between January 1985 and December 2015 as part of their treatment for neoplastic or haematological disease were studied. Preoperative variables, including underlying diagnoses, signs, symptoms, radiology and laboratory data, and transfusion information, were recorded in a prospectively developed database. Karnofsky performance status^19^ was measured before and 1 month after surgery.

Clinical and haematological information was recorded at 6 months, 1 year and 2 years. Long‐term follow‐up was also available for most patients. Informed consent for surgery and data collection was obtained from each patient.

**Figure 1:**
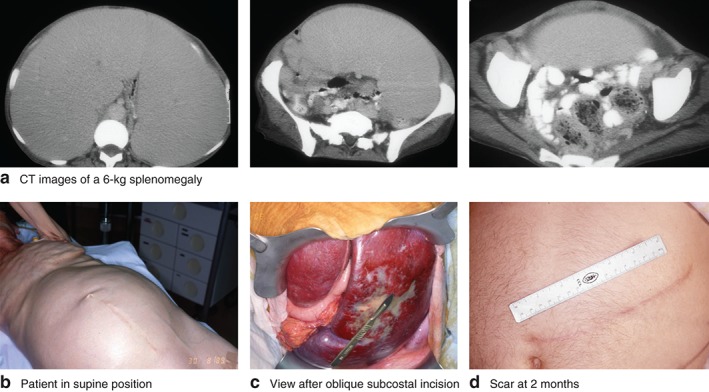
**a** CT images of a 6·0‐kg splenomegaly extending down to the pelvis in a cachectic female patient (bodyweight 36 kg, BMI 12 kg/m^2^) presenting with myelofibrosis. **b** The patient was placed in the supine position, turned slightly to the right. **c** Intraoperative view after oblique subcostal incision. **d** A 15‐cm scar seen 2 months after surgery

Splenectomy was generally planned as an open procedure, predominantly through an oblique incision starting below the left costal margin at the level and in the axis of the ninth intercostal space and descending towards the umbilicus (*Fig*. 1). In a small number of patients, a laparoscopic approach was considered at the outset. The patient was in the supine position, turned slightly to the right, and strapped to the operating table to allow for steep changes in table position. After mobilization of the lower part of the spleen from the peritoneal cavity, the gastrosplenic ligament was divided widely to gain early access to the main splenic artery above the pancreatic tail. Early ligation of the splenic artery permitted some decompression of very large spleens by transfusing the contents before ligation of the venous return. All spleens were weighed within minutes of excision. All patients received standard preoperative antibiotic prophylaxis, and were immunized against encapsulated bacteria by receiving preoperative polysaccharide vaccines against *Streptococcus pneumoniae, Haemophilus influenzae* and *Neisseria meningitidis* at least 3 weeks before elective splenectomy.

Prophylactic measures to reduce the risk of venous thromboembolism included early mobilization, antiembolism stockings and administration of low‐dose low molecular weight heparin. Aspirin (100 mg/day) was used for at least 6 months in patients with reactive thrombocytosis (platelet count above 500 × 10^9^/l).

For comparison of spleen weight and bodyweight/spleen weight ratio, patients were divided into four groups: those with non‐Hodgkin's lymphoma, myeloid dysplasia, chronic lymphocytic leukaemia and rare diseases.

### Statistical analysis

All data are displayed as means or medians with ranges depending on data distribution for continuous variables, and as frequency or percentages for categorical variables. When indicated, categorical variables were compared using the χ^2^ test. Kaplan–Meier analysis was undertaken to estimate the postoperative cumulative survival probability for the whole series.

As the persistence of a favourable outcome for pain relief, control of anaemia and thrombocytopenia could be biased by patient death during long‐term follow‐up, the analysis considered the effects of death during follow‐up as a censoring or competing risk event on the inference made for these three outcomes^20^.

Bodyweight and height were measured to derive the BMI, and the bodyweight/spleen weight ratio was correlated with BMI to take the patient's morphology into account. As there was a common component (bodyweight), mathematical coupling of data was considered and also taken into account^21^.

## Results

Baseline characteristics of the 56 patients are summarized in *Table* 1 and pathological diagnoses in *Table* 2. More than two‐thirds (71 per cent) of the patients were 60 years of age or older. With one exception of idiopathic left‐sided portal hypertension, all patients had some form of haematological disease. Mean duration of disease before surgical involvement was 60 (median 24, range 9–164) months. Spleen weights and bodyweight/spleen weight ratios for the entire series and for each of the four disease groups are shown in *Table* 1.

**Table 1:** Baseline patient characteristics

	No. of patients (*n* = 56)*
Age (years)†	65 (28–83)
Sex ratio (M : F)	32 : 24
Bodyweight (kg)†	64 (36–140)
BMI (kg/m^2^)†	23 (12–43)
ASA fitness grade	
I	1 (2)
II	18 (32)
III	33 (59)
IV	4 (7)
Co‐morbidity (≥ 2)	31 (55)
Other abdominal surgery at time of splenectomy	10 (18)
Preoperative chemotherapy	32 (57)
Preoperative steroid treatment	16 (29)
Preoperative transfusion of red cells	26 (46)
Spleen weight for entire series (kg)†	2·3 (1·5–6·0)
Non‐Hodgkin's lymphoma (*n* = 27)	2·1 (1·5–5·0)
Myeloid metaplasia (*n* = 15)	2·8 (1·8–6·0)
Chronic lymphocytic leukaemia (*n* = 7)	2·3 (1·7–3·5)
Other (*n* = 7)	1·6 (1·5–2·5)
Bodyweight/spleen weight ratio†	29 (6–66)
Non‐Hodgkin's lymphoma (*n* = 27)	34 (12–48)
Myeloid metaplasia (*n* = 15)	25 (6–36)
Chronic lymphocytic leukaemia (*n* = 7)	23 (16–52)
Other (*n* = 7)	48 (28–66)

* Unless indicated otherwise;

† values are median (range).

**Table 2:** Pathological diagnoses in 56 patients with massive splenomegaly

	No. of patients
Non‐Hodgkin's lymphoma	27
Polycythaemia vera	6
Myelofibrosis	9
Chronic lymphocytic leukaemia	4
Hairy cell leukaemia	3
Microspherocytosis	2
Niemann–Pick disease	1
Histiocytic sarcoma	1
Immune thrombocytopenic purpura in Hodgkin's disease	1
Idiopathic left‐sided portal hypertension	1
Sideroplastic anaemia with haemochromatosis	1

**Figure 2:**
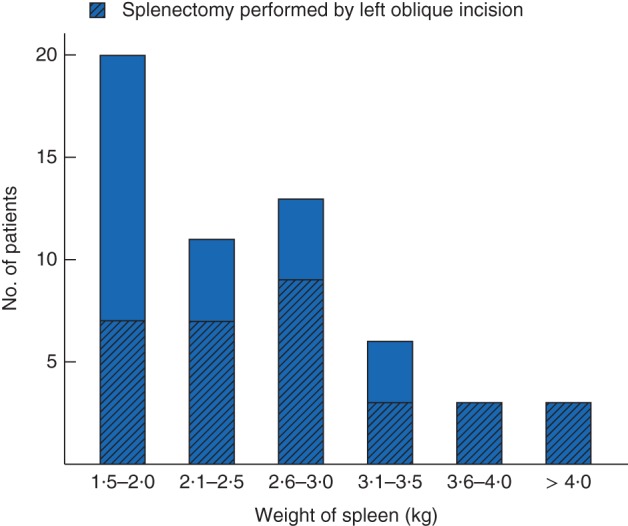
Dry weight distribution of the 56 removed spleens. Hatched areas indicate the 32 patients operated on by the left oblique incision

**Figure 3:**
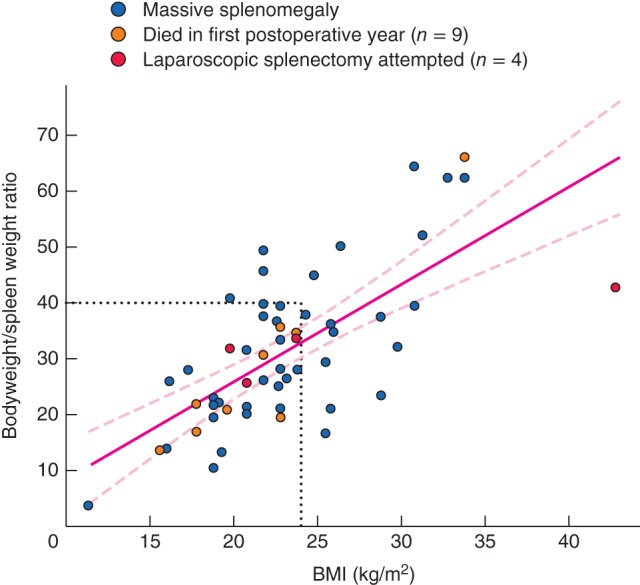
Scatter plot of bodyweight/spleen weight ratio against BMI in 56 patients with massive splenomegaly. The left lower quadrant delineated by dotted lines (BMI less than 24 kg/m^2^ and bodyweight/spleen weight ratio below 40) includes 35 of the 42 patients who had excellent pain relief after splenectomy (Karnofsky performance score increased from 20–50 to a postoperative score of 70–90). The regression line with its 95 per cent c.i. is shown. R = 0·69, P < 0·001

The mean duration of surgery was 128 min, and ten patients (18 per cent) required intraoperative blood transfusion (2–5 units of packed red cells). Platelet concentrate was generally infused after early ligation of the splenic artery in those with severe preoperative thrombocytopenia. The weight distribution of the removed spleens is shown in *Fig*. 2; 12 patients had a spleen weight greater than 3·0 (mean 3·2; median 3·4, range 3·1–6·0) kg. The scatter plot of bodyweight/spleen weight ratio against BMI with the corresponding regression line is shown in *Fig*. 3.

Open splenectomy was performed in 55 patients; hand‐assisted laparoscopic splenectomy was attempted in four patients, with conversion to open surgery in three of these. The oblique left upper quadrant incision was used in 32 patients (57 per cent), mainly for spleens weighing more than 2·5 kg. A subcostal incision was used in 13 patients (23 per cent). Of 30 patients (54 per cent) who had early intraoperative ligation of the main splenic artery, none required intraoperative or postoperative blood transfusion.

**Figure 4:**
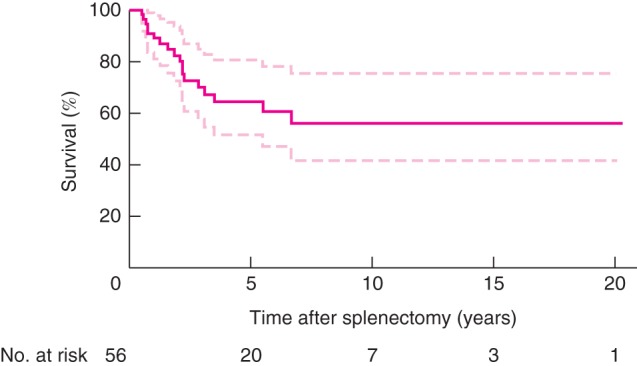
Kaplan–Meier survival estimate with 95 per cent c.i. for 56 severely compromised patients with massive splenomegaly

All 56 patients were discharged from hospital, and there was no death within 180 days of operation. Mean duration of stay from surgery to hospital discharge was 12 (median 10, range 5–21) days. *Fig*. 4 shows Kaplan–Meier survival estimate with 95 per cent c.i.

A total of 17 postoperative complications occurred in 14 of the 56 patients (25 per cent): eight chest infections that responded rapidly to treatment; four pleural effusions (1 required drainage); two cases of early bleeding from the splenic bed that required surgical operation; one incisional hernia after median laparotomy; one case of severe epistaxis in a patient with chronic lymphocytic leukaemia and severe preoperative thrombocytopenia (platelet count 2·8 × 10^9^/l); and one portal vein thrombosis.

Factors that were significantly associated with postoperative complications were ASA grade above II (P = 0·015) and bodyweight/spleen weight ratio less than 20 (P = 0·044) (Table 3). Of 37 patients classified as ASA grade III or IV, 13 developed postoperative complications, compared with only one of the 19 patients with ASA grade I or II. The use of short‐term high‐dose steroids, prescribed before surgery in 16 patients (29 per cent) as part of the medical management of their haematological disorder, was not associated with postoperative complications when compared with patients who had not received steroids.

**Table 3:** Factors significantly associated with postoperative complications

	No. of complications	P *
Preoperative need for blood transfusion		0·001
Yes	12 of 26	
No	2 of 30	
Presence of ≥ 2 co‐morbidities		0·001
Yes	13 of 31	
No	1 of 25	
ASA fitness grade > II		0·015
Yes	13 of 37	
No	1 of 19	
Bodyweight/spleen weight ratio < 20		0·044
Yes	5 of 10	
No	9 of 46	

* χ^2^ test.

**Figure 5:**
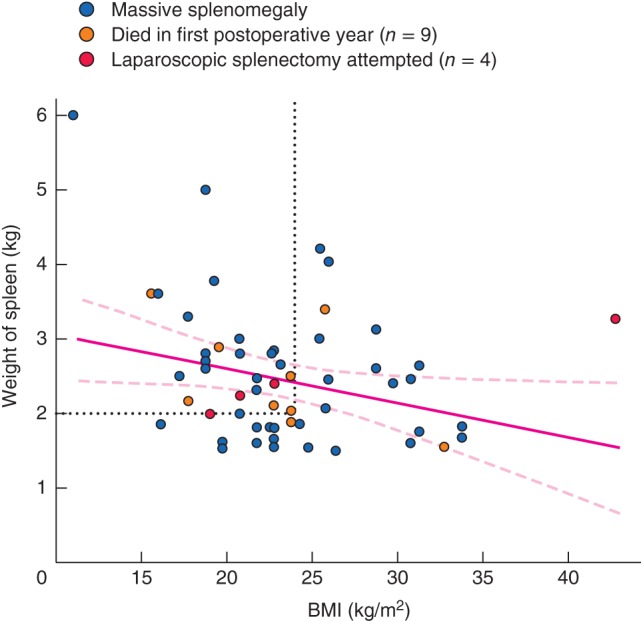
Comparison of spleen weights with BMI, performed to eliminate the possibility of superimposed mathematical coupling. In the left upper quadrant delineated by dotted lines (BMI less than 24 kg/m^2^ and spleen weight above 2·0 kg) are the 25 patients who had excellent pain relief after splenectomy, as assessed by the Karnofsky performance score. The regression line with its 95 per cent c.i. is shown. R = −0·28, P = 0·036

Palliation was effective in 42 patients in whom splenectomy was performed primarily for pain relief. Analysis of BMI and the bodyweight/spleen weight ratio indicated that 35 of these 42 patients had excellent pain relief, and moved from preoperative Karnofsky performance scores of 20–50 to postoperative scores of 70–90 (Fig. 3). To overcome the problem of mathematical coupling, spleen weight alone was compared with BMI (Fig. 5). Spleen weight correlated negatively with BMI. This still allowed the identification of 25 patients who moved from a Karnofsky score of 20–50 to a score of 70–90 after splenectomy. The provisional cut‐off points adopted for good pain relief were bodyweight/spleen weight ratio below 40, spleen weight greater than 2·0 kg, and BMI below 24 kg/m^2^. Determination of these provisional cut‐off points was based on the tentative delineation of the quadrant of the scatter plot encompassing a maximum of patients who had good pain relief in the face of a low preoperative bodyweight/spleen weight ratio. In addition, nine patients who died during the first postoperative year were located in the same quadrant.

After censoring for death, relief of pain was maintained in 33 of 34 patients at 2 years (*Table* 4). Anaemia and thrombocytopenia resolved at 6 months either partially (requiring less transfusion) or completely in 15 of 22 (68 per cent) and 15 of 16 (94 per cent). At 2 years, sustainable independence from red blood cell transfusion was maintained in 15 of 19 patients who had anaemia before surgery, and from platelet transfusion in nine of 11 patients with preoperative thrombocytopenia (*Table* 4).

**Table 4:** Death‐censored outcomes following splenectomy

	Time after splenectomy		
	6 months	1 year	2 years
Relief of volume–pressure symptoms	40 of 42	37 of 39	33 of 34
Control of anaemia	15 of 22	16 of 21	15 of 19
Control of thrombocytopenia	15 of 16	11 of 12	9 of 11

Reactive thrombocytosis (platelet count above 500 × 10^9^/l) occurred in 27 of the 56 patients (48 per cent), of whom 16 had been thrombocytopenic before splenectomy. Thrombocytosis resolved within 3–9 months, with no occurrence of deep venous thrombosis. These 27 patients had received aspirin (100 mg/day) for at least 6 months before surgery. A single patient with myelofibrosis developed portal vein thrombosis, diagnosed 2 months after splenectomy, and died 8 months after the operation.

## Discussion

Experience with splenectomy for massive splenomegaly is often limited because this operation concerns only a small proportion of patients who undergo splenectomy as treatment for their haematological disease^2^, ^3^, ^4^, ^5^, ^6^, ^7^. Laparotomy is usually required to remove a massive spleen. Pioneers of laparoscopic splenectomy^11^, ^12^ concluded that spleens weighing more than 3·2 kg required conversion to open surgery. Others^22^, ^23^ have suggested a limitation of a splenic size greater than 27 or 30 cm.

In the present study, there was no correlation between the incidence of complications and age, preoperative chemotherapy, steroid treatment, volume and/or weight of the spleen, nature of the underlying disease or intraoperative blood loss. These findings differ from those of at least one other study^6^, which concluded that increased age and underlying illness were the predominant factors associated with morbidity and mortality following splenectomy for massive splenomegaly. In another study^7^, the preoperative categorical variables that significantly correlated with postoperative complications were preoperative need for blood transfusion and the presence of more than two co‐morbidities.

The zero mortality rate achieved in the present study was also shown in 1987 by Bickerstaff and Morris^2^. Complications after splenectomy for massive splenomegaly include bleeding, respiratory infection, subphrenic abscess, and splenic or portal vein thrombosis^8^, ^24^, ^25^, ^26^, especially in patients with myeloproliferative disorders^24^. In a large series^8^ published in 2013 involving 222 consecutive patients, the 30‐day mortality rate was 1·8 per cent and the complication rate 20 per cent. The most common complications were bleeding (9 per cent) and portal venous thrombosis (9·9 per cent), as detected by postoperative imaging^8^. This rate suggests that postoperative portal vein thrombosis is underestimated because postoperative imaging is not performed routinely, being reserved for patients with persistent symptoms^8^, ^27^. If there were other patients in the present series with portal vein thrombosis, they were not symptomatic.

There is little evidence that splenectomy has an impact on subsequent thromboembolic complications. Reactive thrombocytosis occurred in 27 patients (48 per cent) in the present series. Administration of antiplatelet agents such as aspirin for prevention of complications related to postsplenectomy thrombocytosis may be of benefit in patients presenting with additional cardiovascular risk factors^26^, ^27^, ^28^. General prophylactic measures such as early mobilization and antiembolism stockings combined with low‐dose low molecular weight heparin are also indicated to reduce the risk of thromboembolism^26^. Policy in the present series was routine application of these measures in all patients during the perioperative period, and to continue aspirin (100 mg daily) for at least 6 months if reactive thrombocytosis (platelet count above 500 × 10^9^/l) occurred.

The use of short‐term high‐dose steroids as part of the medical management of haematological disorders did not constitute a potentially higher risk of postoperative complications. This is in line with the results of a previous study^29^, which showed that chronic bronchopulmonary disorders requiring a long duration (median 24 months) of steroid therapy at low doses (median 0·51 mg per kg per day of hydrocortisone or equivalent) were associated with a greater risk of postoperative steroid‐related complications than haematological disorders requiring a shorter duration of steroid treatment (median 6 months) at higher doses (median 1·20 mg per kg per day).

Splenectomy appeared to offer durable palliation for the correction of haematological cytopenia in the present study, as shown in earlier reports^2^, ^6^, ^7^, ^8^. Symptom palliation and need for transfusion were excellent in the present series, along with advantageous survival in terms of both duration and performance status. The threshold to perform splenectomy for massive splenomegaly should remain low, because the benefits are significant for the majority of patients. Janus kinase 2 inhibitors are new potential therapeutic options for splenomegaly associated with primary or secondary myelofibrosis^30^, ^31^. If these inhibitors reduce the size of the spleen and improve quality of life, the need for splenectomy could diminish, along with the risk of portal vein thrombosis.

The literature relating to splenic surgery contains an inconsistent terminology that seems to have worsened with the introduction of laparoscopic surgery. In addition, subsets of spleens weighing 600–1600 g or more have been classified as massive or supramassive^15^, ^16^. This problem might be overcome with the wider assessment of splenic size by three‐dimensional reconstruction of CT images to calculate spleen volume.

Adjustment of spleen size and weight to the patient's morphology and habitus seems a logical step that might influence the surgical decision‐making process, such as the use of a laparoscopic approach. The scatter plot of bodyweight/spleen weight ratios *versus* BMI demonstrated that the lower the ratio, the lower the BMI. In general the very large spleen is more likely to cause anorexia and weight loss. In this study, a bodyweight/spleen weight ratio below 20 was significantly associated with the occurrence of postoperative complications (*P* = 0·044).

The relationship between bodyweight/spleen weight ratio and BMI was further explored by comparing spleen weight alone with BMI to overcome mathematical coupling. The relationship was still significant. This relationship was also effective in identifying 60 per cent of patients (25 of 42) who had excellent pain relief, confirmed by an improvement in their Karnofsky performance score^19^, ^32^. The provisional cut‐off points for good pain relief are spleen weight greater than 2·0 kg and BMI below 24 kg/m^2^. In general, the implication is that the larger the spleen, the greater the improvement following splenectomy.

The adjustment of spleen weight and volume (by preoperative 3‐dimensional CT reconstruction) to BMI could offer a potentially useful estimate of a patient's preoperative condition and morphology. This should be confirmed by independent studies, in the hope of defining practical cut‐off points for BMI and spleen weight that could inform surgical decision‐making. These data could lead to a more practical definition of massive splenomegaly rather than a simple weight of more than 1·5 kg, a measurement that is only really available after operation.

## Disclosure

The authors declare no conflict of interest.
